# The Parent-Adolescent Relationship and Risk-Taking Behaviors Among Chinese Adolescents: The Moderating Role of Self-Control

**DOI:** 10.3389/fpsyg.2019.00542

**Published:** 2019-03-20

**Authors:** Lu Liu, Na Wang, Lumei Tian

**Affiliations:** School of Psychology, Shandong Normal University, Jinan, China

**Keywords:** Chinese adolescents, parent-adolescent conflict, perceived parental support, risk-taking behaviors, self-control

## Abstract

The present study primarily aimed to examine whether self-control serves as a moderator in the associations between parent-adolescent relationships, including parental support and parent-adolescent conflict, and risk-taking behaviors among adolescents. The 917 Chinese adolescents whose mean age was 14.38 years (*SD* = 1.69) completed questionnaires effectively. The results indicated that the relationships between either parental support or parent-adolescent conflict and adolescent risk-taking behavior were moderated by self-control. Among those adolescents with lower levels of self-control, both higher levels of parent-adolescent conflict and lower levels of perceived parental support predicted more risk-taking behaviors, but their predicting roles got weakened with the increase of the level of self-control. Accordingly, good parent-adolescent relationship, particularly less parent-adolescent conflict, is critical for decreasing adolescent risk-taking. Otherwise, improving self-control is particularly helpful to those adolescents having more conflict with their parents or less parental support to decrease their risk-taking.

## Introduction

Risk-taking behaviors refers to participating in behaviors which probably lead to some aversive consequences (Boyer, 2006). In general, it includes negative risk-taking behaviors, sometimes called problem behaviors, and positive risk-taking behaviors that is challenging but relatively socially acceptable (Özmen and Sümer, 2011). The former includes smoking, binge drinking, drug use and unsafe sexual behaviors, and so on, whereas the latter includes bungee jumping, skiing, and diving and other risky sports. During the last decade, scholars have paid much attention to negative risk-taking behaviors, such as alcohol use (O’Hara and Cooper, 2015; Passos et al., 2015), substance use (Jacobus et al., 2013; Wood et al., 2013), sexual risk taking (Downing and Bellis, 2009; O’Hara et al., 2012) and so on, because negative risk-taking behaviors not only do harm to adolescents’ physical development but also have adverse impacts on their psychosocial adaptation. For comparison, the current study also put emphasis on negative risk-taking behaviors and tried to find out those risk factors or buffers for adolescent negative risk-taking behaviors. We did this job according to Problem-Behaviors Theory (PBT; Jessor, 1987), a psychosocial perspective focusing on the Personality System, the Environment System, and their interaction, which believes that individual behavioral performance needs to be studied from the perspective of development, as well as the interactions between the three systems.

Self-control is an important factor in the Personality System, which is defined as the ability of human beings to suppress automatic, habitual or innate behaviors, impulsiveness, emotion, or desire, otherwise these behaviors will interfere with target oriented behaviors (Muraven et al., 2006), which glasses a struggle between urges, desires, and inhibitory forces (Hofmann and Van Dillen, 2012). That is to say, individuals with high self-control are theorized to have abilities to inhibit impulses and resist immediate pleasures (Hay and Forrest, 2008), while individuals with low self-control including traits like urges and feeling seeking (Doran et al., 2011) are thought to be the opposite. Meanwhile, the role of self-control in adolescent adjustments has obtained much attention empirically. For example, higher level of self-control is correlated with better physical and mental health (Tu and Yang, 2016); increased self-control is linked to decreased aggression (Keatley et al., 2017) and reduced involvement in gambling behaviors among adolescents (Belle and Tammie, 2015); there are significant negative correlations between self-control and school violence (Agbaria and Daher, 2015; Zimmerman et al., 2015); those adolescents whose impulse control deteriorated during secondary school also had a higher risk of using substances during high school (James et al., 2016). Accordingly, the current study also took self-control into great account linked with adolescent risk-taking behaviors.

One of contextual factors that may have great influences on adolescents is family. Of particularly important is parent-adolescent relationship. As a positive feature, parental support for basic psychological needs has a positive relationship with the Psychosocial adaptation of children and adolescents (Tu et al., 2016). Parents mostly provide emotional and instrumental support for adolescents and build psychological connections with them (Shakespeare-Finch and Obst, 2011), and emotional support is believed to promote children’s internalization of social values and criterions (Patterson et al., 1989), making children more sensitive to social hints before action. Instrumental support is linked to practical assistance, playing a prominent role in offering advice and guidance to their children (Cheung and Sim, 2014). For example, Padilla-Walker et al. (2016) found that parental warmth was positively related to adolescents’ pro-social behaviors; low instrumental support was associated with lower self-perception and higher level of depression (Wang et al., 2018), and there are negative associations between perceived parental support and behaviors problems (Yun et al., 2016), such as alcohol use (Maslowsky et al., 2016). Similarly, an inverse relationship has been found between perceived familial support and cigarette smoking (Hamid et al., 2015). However, the tendency of adolescents to think that parents are absolutely authoritative is declining (Fuligni and Eccles, 1993) and adolescents demand more autonomy (Laursen and Collins, 2004), leading to an increase in conflicts with parents (Steinberg and Morris, 2001). As Atkinson et al. (2005) put forward, when conflict took place during family discussions or communications, one’s emotional circuits in the brain were stimulated at the same time and it became increasingly difficult for an individual to reset his or her mind. As a result, their attention to information of externalizing behaviors is not enough. Considerable research has revealed the relationship between conflict and negative consequences for adolescents. For example, it has been found that conflicts between parents and adolescents are related to antisocial behaviors (Sentse and Laird, 2010), alcohol use (Chaplin et al., 2012; Abar et al., 2014), etc. Note that positive features and negative features of relationships are independent, not opposite ends of a continuum (Laursen and Mooney, 2008), independently predict adolescent externalizing problems, internalizing problems, and academic achievement (Adams and Laursen, 2007). Thus, it is important to focus on the effects of the two aspects of parent-adolescent relationship, support, and conflict, simultaneously, on adolescent adjustments.

Based on PBT, when the proneness in the Personality System and the Environment System is taken together, the combination may generate psychosocial tendency to make the prediction and explanation of problem behaviors. For example, positive emotionality can act as a resilience effect of a connection between parent-child clash and material use of adolescents (Wills and Sandy, 2001). Also, the associations of perceived parental support with aggressive or delinquent behaviors tend to be negative for adolescents with high empathy but positive for those with low empathy (Van der Graaff et al., 2012). Among persons with good self-control, risk factors (such as negative life events) have a reduced impact on drug use (Wills et al., 2008). As an individual’s self-control increases, they require less social support to achieve subjective well-being; however, those with lower levels of self-control experience the opposite (Zhang and Xing, 2007). It can be seen that self-control can moderate the relationship between environmental factors and social adaptation. However, work examining whether adolescents varying in self-control are differentially influenced by parent-adolescent relationship, especially on adolescent risk-taking behaviors, is really limited. To our knowledge, no studies have tested whether self-control moderates the association between parent-adolescent relationship and risk-taking behaviors among adolescents. Therefore, in consistent with research indicating that low studious control and conflict between parents and adolescents is a common risk factor for depression, as well as participating in antisocial behaviors in adolescents (Wang et al., 2013), parent-adolescent conflict and low level of self-control in the current study are also expected as risk factors for adolescent risk-taking behaviors. Also, according to the risk-enhancing model (Fergus and Zimmerman, 2005), low self-control, as a risky factor, increases risks of low level of parental support. Accordingly, we hypothesized that low level of perceived parental support and high level of parent-adolescent conflict would increase adolescent risk-taking behaviors at low level of self-control while not at high level of self-control.

Lastly, note that adolescent risk-taking behaviors might predict parent-adolescent relationship as well. Transactional model provides theoretical support for this possibility, which holds that development is the result of a sustained and dynamic two-way interaction between individuals and their environmental experience (Sameroff and Mackenzie, 2003). A follow-up study on adolescents found that internalizing and externalizing problem behaviors in the pre-test significantly predicted parenting attitudes and behaviors in the post-test (Buist et al., 2004). A cross-lagged panel model also revealed a reciprocal association between mother-adolescent relationship quality and adolescent antisocial behaviors (Crocetti et al., 2016). Accordingly, we established a competitive model in which adolescent risk-taking behaviors were the predictor, while parent-adolescent relationship was the outcome and hypothesized that more risk-taking behaviors would lead to poorer parent-adolescent relationship.

## Materials and Methods

### Participants

The participants were recruited from a public secondary school and a public high school which were located in a county in Shandong Province in eastern China. They were all from rural areas. A total of 980 adolescents were invited as the initial sample. Among them, 917 adolescents completed the questionnaires, giving a response rate of 93.6%. Therefore, the final sample consisted of 917 Chinese adolescent students from 11 to 19 years old (mean age = 14.38, *SD* = 1.69; 493 boys).

### Measures

#### Parent-Adolescent Relationship

Perceived parental support or parent-adolescent conflict was measured using the Chinese shortened version (Tian et al., 2012) of the Network of Relationships Inventory (NRI; Furman and Buhrmester, 1985). It includes 15 items in both relationships with father and mother and is made up of five dimensions, including companionships (e.g., “How long are you with this person when you are free?”), instrumental help (e.g., “Does the person often give you help when in need?”), affection (e.g., “How much does the person like you?”), intimacy (e.g., “Do you share your secrets or feelings with the person?”), and conflict (e.g., “Is there much quarrel between your parent and you?”), each dimension consisting of three items. For each item, participants were asked to rate it on a 5-point Likert scale, ranging from 1 (little or none) to 5 (the most). Because the former four dimensions correlated highly with each other and correlations between the mother’s and the father’s scores were high as well (0.46 < *rs* < 0.81, *p* < 0.001) in the present study, scores of them were combined into a composite parental support score, according to the procedure of previous research (Furman et al., 2002; Rubin et al., 2004; Tian et al., 2012, 2014). With higher scores, the levels of parental support are higher, and its Cronbach’s alpha coefficient was 0.94. Similarly, we averaged the scores on mothers and fathers to compose one score for parent-adolescent conflict. The higher the score, the more serious the conflict between parents and adolescents, and its Cronbach’s alpha coefficient was 0.90. The Cronbach’s alpha coefficient for the whole NRI was 0.91 in the present study. The fit indices from a confirmatory factor analysis were adequate, χ^2^/df = 2.80, GFI = 0.97, TLI = 0.98, CFI = 0.98, and RMSEA = 0.04.

#### Self-Control

The Self-Control Scale for Chinese middle and high school students (SCS) was developed for assessing the ability of self-control of participants in learning, entertainment, and social life (Wang and Lu, 2004). It consists of 36 items (e.g., “I will shout and yell when I am happy” or “I am easily influenced by the outside”). For each statement, participants were asked to rate it on a 5-point Likert scale, ranging from 1 (completely disagree) to 5 (completely agree). With higher scores, the levels of self-control are higher. In the present study, the Cronbach^’^s alpha coefficient of the SCS was 0.88. The fit indices from a confirmatory factor analysis were adequate, χ^2^/df = 2.80, GFI = 0.91, TLI = 0.88, CFI = 0.90, and RMSEA = 0.04.

#### Risk-Taking Behaviors

The Chinese version (Zhang et al., 2011) of the Adolescent Risk-Taking Questionnaires (ARQ; Gullone et al., 2000) was used to measure the frequency of participation in each of some behaviors (on a 5-point Likert scale: 0 = never done, 4 = done very often). It consists of 17 items with a satisfactory reliability and validity that can be applied to the assessment of Chinese adolescent risk-taking behaviors (Zhang et al., 2011). It is comprised of four dimensions, including thrill-seeking behaviors (five items, e.g., “going skating” or “going skiing”), reckless behaviors (two items, e.g., “drug abuse” or “unsafe sexy behaviors”), rebellious behaviors (six items, e.g., “smoking” or “alcohol use”), and antisocial behaviors (four items, e.g., “making fun of others” or “having cheated on school tests”). Thrill-seeking behaviors are regarded as positive or socially acceptable behaviors, while the last three dimensions are regarded as conveying negative risk-taking behaviors (Moore et al., 2004). Thus the last 3 dimensions were only investigated in the current study, and a risk-taking behaviors total score was calculated for each adolescent with a high score indicating more risk-taking behaviors. The Cronbach^’^s alpha coefficient was 0.71. The fit indices from a confirmatory factor analysis were adequate, χ^2^/df = 3.22, GFI = 0.96, TLI = 0.94, CFI = 0.95, and RMSEA = 0.05.

### Procedure

In current research, data were collected by trained graduate students in participants’ respective classrooms. Before survey, participants were informed that the study was anonymous and they could withdraw freely. The present study adopted a passive consent procedure recommended by Ellickson (1989), which requires students and their parents to return forms only when they do not want to participate in the study. Those who do not return the forms are assumed to agree to participate in the study. Then, all participants were given a series of self-reported questionnaires, and all measures were completed in approximately 20 min. Ethics approval was obtained from the Research Ethics Boards at our university.

### Statistical Analysis

After obtaining the descriptive statistics for the study variables and assessing the correlations among them, separate hierarchical linear regression analyses were conducted using SPSS Version 22 to examine the hypotheses. Gender was re-coded into dummy variables separately (0 = male, 1 = female). The scores on perceived parental support, parent-adolescent conflict, and self-control were all transformed into Z scores before creating the interaction items (Aiken and West, 1991). In the regression analysis, the predictors were entered in three hierarchical steps: (1) age and gender; (2) perceived parental support or parent-adolescent conflict and self-control; and (3) the two-way interaction item. Post-hoc probing of significant interactions was conducted using the simple slope analysis (Aiken and West, 1991) when the predictor criterion was examined at high (i.e., 1 standard deviation above the mean) and low (i.e., 1 standard deviation below the mean) levels of self-control.

## Results

### Common Method Biases

The common-method bias might occur owing to all data in the present study deriving from adolescents’ self-reports. Thus, when collecting the data, we made questionnaires anonymous and made some items reverse scoring. Prior to data analysis, Harman’s one-factor test was conducted, in which 17 factors with one-above Eigen values were extracted; in addition, the first factor could explain 17.28% of the variance much lower than the critical value of 40%. Therefore, there was no serious common-method bias in the current study.

## Descriptive Statistics and Correlation Analysis

Table 1 gives the means, standard deviations, and correlations of the study variables. Perceived parental support and self-control were negatively associated with risk-taking behaviors (*r* = −0.11, *p* < 0.001; *r* = −0.40, *p* < 0.001), whereas parent-adolescent conflict was positively correlated with risk-taking behaviors (*r* = −0.16, *p* < 0.001). The effects of gender (*t* (915) = 4.00, *p* < 0.001) and age (*r* = 0.25, *p* < 0.001) on risk-taking behaviors were both significant and thus were controlled statistically in the next analyses.

**Table 1:** Descriptive statistics and correlations among study variables (*N* = 917).

Variables	*M*	*SD*	1	2	3
1. Perceived parental support	3.36	0.78	一		
2. Parent-adolescent conflict	2.02	0.82	−0.16^***^	一	
3. Self-control	3.30	0.48	0.26^***^	−0.45^***^	一
4. Risk-taking behaviors	0.35	0.39	−0.11^**^	0.27^***^	−0.40^***^

*Note:* ^***^ *p* < 0.001, ^**^ *p* < 0.01, ^*^ *p* < 0.05.

## Testing the Moderation Models

**Figure 1:**
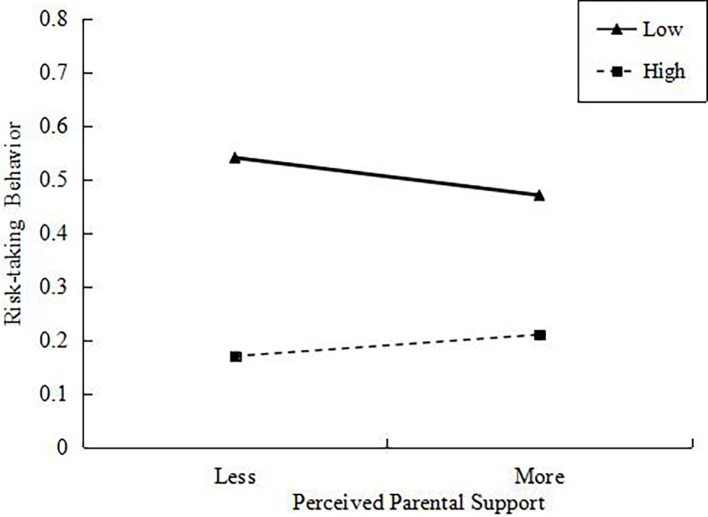
Interaction between perceived parental support and self-control on adolescent risk-taking behaviors.

As shown as in Table 2, there was a negative main effect of self-control and a significant interaction between perceived parental support and self-control on adolescent risk-taking behaviors. Simple slope analyses (See Figure 1) indicated that perceived parent support was associated with risk-taking behaviors marginally significant at low levels of self-control (*b_simple slope_* = −0.04, *t =* −1.68, *p =* 0.09), but the association was not significant statistically at high levels of self-control (*b_simple slope_* = 0.03, *t =* 1.29, *p* = 0.20).

**Table 2:** Hierarchical regression analysis for perceived parental support and self-control predicting risk-taking behaviors.

	Predictors	*ΔR* ^2^	*β*	*t*	95%CI
Step 1	Age	0.08	0.06	7.62^***^	[0.04, 0.07]
	Gender		−0.10	−4.03^***^	[−0.15, −0.05]
Step 2	A: Perceived parental support	0.13	−0.01	−0.76	[−0.03, 0.02]
	B: Self-control		−0.14	−11.61^***^	[−0.17, −0.12]
Step 3	A × B	0.01	0.03	2.72^**^	[0.01, 0.05]

*Note:* ^***^ *p* < 0.001, ^**^ *p* < 0.01, ^*^ *p* < 0.05.

**Figure 2:**
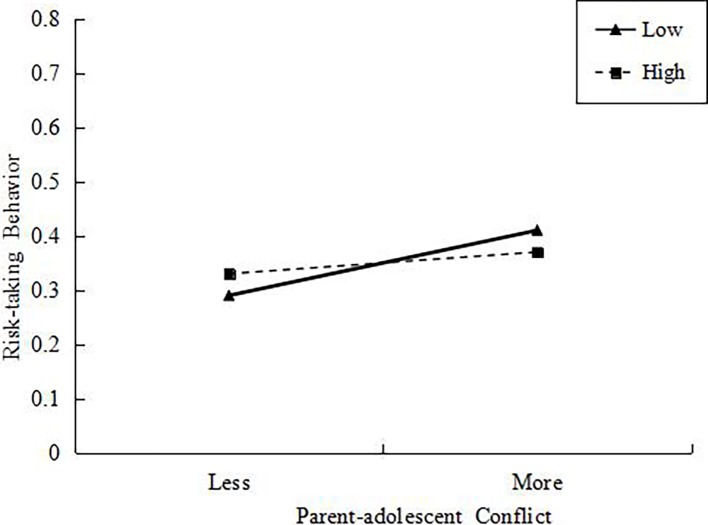
Interaction between parent-adolescent conflict and self-control on adolescent risk-taking behaviors.

As shown as in Table 3, however, there were a positive main effect of parent-adolescent conflict on risk-taking behaviors and a negative main effect of self-control on risk-taking behaviors, as well as the significant interaction between them. Simple slope analyses (See Figure 2) indicated that among those adolescents with low levels of self-control, greater levels of parent-adolescent conflict predicted more risk-taking behaviors (*b_simple slope_* = 0.07, *t* = 3.63, *p* < 0.001), but the association was not significant statistically among those with high levels of self-control (*b_simple slope_* = 0.03, *t* = 1.39, *p* = 0.16).

**Table 3:** Hierarchical regression analysis for parent-adolescent conflict and self-control predicting risk-taking behaviors.

	Predictors	*ΔR* ^2^	*β*	*t*	95%CI
Step 1	Age	0.08	0.06	7.61^***^	[0.04, 0.07]
	Gender		−0.10	−4.03^***^	[−0.15, −0.05]
Step 2	A: Parent-adolescent conflict	0.15	0.05	4.00^***^	[0.03, 0.08]
	B: Self-control		−0.12	−9.39^***^	[−0.15, −0.10]
Step 3	A × B	0.01	−0.02	−2.31^*^	[−0.05, −0.004]

*Note:* ^***^ *p* < 0.001, ^**^ *p* < 0.01, ^*^ *p* < 0.05.

To test the competitive model, we also used regression analysis, in which risk-taking behaviors was the independent variable and parent-adolescent relationship was the outcome variable. The analyses indicated that there was a negative main effect of risk-taking behaviors on perceived parental support and a positive main effect on parent-adolescent conflict (*β* = −0.10, *p* < 0.001, *R^2^* = 0.02; *β* = 0.23, *p* < 0.001, *R^2^* = 0.09, respectively), but their effect sizes (*R^2^*) were much smaller than those in the hypothesized model (*R^2^* = 0.09, *R^2^* = 0.15). The interaction between risk-taking behaviors and self-control was not obvious (*p* = 0.65; *p =* 0.12), which indicated that the competitive moderation model was not well established.

## Discussion

The main goal of the present study was to examine the associations of parent-adolescent relationships, including parental support and parent-adolescent conflict, with adolescents risk-taking behaviors and the moderating role of self-control. Overall, the results indicated that for those adolescents with lower levels of self-control, both higher levels of parent-adolescent conflict and lower levels of perceived parental support predicted more risk-taking behaviors, but their predicting roles got weakened with the increase of the level of self-control. Additionally, parent-adolescent conflict was significantly related to adolescents risk-taking behaviors, whereas the relationship between parental support and risk-taking behaviors was not obvious.

First, in agreement with previous research reporting that increased self-control is linked to decreased aggression (Keatley et al., 2017), self-control was also negatively correlated with adolescent risk-taking behaviors in the present study. This is possibly because adolescents lower in self-control have more traits like impulses and feeling seeking (Doran et al., 2011), which in turn making them more likely to ignore potential passive consequences of their behaviors (Watson and Clark, 1993).

Second, in accordance with research regarding parent-adolescent relationship as important in moulding adolescent mental functioning (Lam et al., 2012; Skinner and Mchale, 2016), high levels of parent-adolescent conflict were associated with more risk-taking behaviors in the present study. When adolescents have more disagreements or conflicts with their parents, emotional circuits in the brain are stimulated (Atkinson et al., 2005), such as causing anger (Chaplin et al., 2012), then easily resulting in heightened risk taking. Chein et al. (2011) have also found that negative environmental factors (e.g., the existence of peers) enlarge adolescents’ ventral striatum activation, resulting in much risk-taking behaviors. Another possible explanation is that high level of parent-child conflict will bring children a negative worldview, which easily causes some externalizing problem behaviors (Vanassche et al., 2014).

Third and importantly, as expected by PBT, the impact of parent-adolescent conflict was qualified by adolescent self-control. Specifically, high levels of parent-adolescent conflict predicted increases in adolescent risk-taking behaviors at low levels of self-control, but this predicting role got weakened at high levels of self-control. This result is in line with previous studies finding that less control and parent-adolescent conflict indicate common risk factors for adolescent depression as well as engagement in misconduct (Wang et al., 2013). Furthermore, it also supports the risk-enhancing model (Fergus and Zimmerman, 2005), suggesting one risky factor will enhance the negative impact of another risky factor on adolescent development and adjustment. However, among those adolescents with high levels of self-control, self-control as a protective factor buffered the impact of parent-adolescent conflict on risk-taking behaviors.

Inconsistent with our expectation, increased perceived parental support was not linked to decreased risk-taking behaviors, even among those adolescents with high levels of self-control. A possible explanation is that low levels of perceived parental support of adolescents, particularly of Chinese adolescents who are usually only one child or have only one sibling in their family because of China’s family planning policy, were not absolutely low (*M* = 3.36 ± 0.78 in the current study) and thereby have no obvious difference in impacting adolescent risk-taking behaviors from those higher levels of perceived parental support. Also, it suggests that parental support is neither a protector nor a risky factor of adolescent risk taking because individuals during adolescence emphasize more on peer support rather than parental support (Macek and Jezek, 2007; Ju et al., 2011). The period of adolescence is in the transition period from school-age children to young adulthood. During this period, students have gradually transferred their main activities from family to social institutions such as schools, classrooms, and juvenile organizations. During this time, the closest relationship with the youth is the peer. At the low levels of self-control, however, lower perceived parent support was associated with more risk-taking behaviors marginally significantly, possibly because that both the factors may have synergistic effects and self-control is a stronger predictor of adolescent risk-taking behaviors than perceived parental support. Therefore, it suggests that in adolescence, it is not enough to reduce risk-taking behaviors only relying on the environment, and that improving the internal personality power of individuals is a more fundamental way.

Finally, consistent with our hypothesis and prior studies (Buist et al., 2004; Crocetti et al., 2016), parent-adolescent relationship and risk-taking behaviors have a reciprocal relationship. However, the effect sizes (*R^2^*) in the hypothesized model were greater than those in the competitive model. Importantly, the competitive moderation model was not well-established. The result provided empirical evidence for the Problem-Behaviors Theory. Parent-adolescent relationship as an important environmental factor has an important impact on adolescent risk-taking behaviors. The more conflict between parents and adolescents, the more risky-taking behaviors adolescents will be taken in their future life (Lam et al., 2012). This conclusion, however, should be accepted carefully before it is further examined, and more supportive evidence should be sought in future studies.

Lastly, there are some limitations to be noted in the present study. First, it was impossible to draw causal conclusions of the relationships between variables because of the cross-sectional design of the current study. Second, given that the particularity of Chinese context, it needs to be cautious to extend the present results to other cultural background. It would be of value to further examine the relationships in other countries or districts. Despite these limitations, the present study may offer a new sight into interaction between self-system and family system on adolescent development and adjustment, and these findings suggest that intervention and prevention measures devoted to decreasing adolescent risk-taking incorporate the interaction of family and personal characteristics.

Despite these limitations, the findings of the current study provide valuable information. First, this study simultaneously examined the moderating roles of self-control in the relationships between both parental support and parent-adolescent conflict with adolescent risk-taking behaviors. The findings of the present study provide evidence for Problem-Behaviors Theory, extend our insight into the mechanisms underlying the associations among parent-adolescent relationships, self-control, and adolescent risk-taking behaviors, and supplement data for previous relevant studies. Second, the current study found that parent-adolescent relationship could predict adolescent risk-taking behaviors and vice versa, which provides further support for transactional model. Finally, the findings may provide guidance for intervention and prevention of adolescent risk-taking behaviors. The study suggests that to decrease adolescents’ risk-taking behaviors we should focus on building good parent-adolescent relationship and improving individual self-control, particularly the latter. It is important for adolescents to boost self-control training, cultivation, development, and promotion.

## Data Availability

All datasets generated for this study are included in the manuscript.

## Author Contributions

LL conducted measurements, data collection, data analysis, and completed the earlier draft of this manuscript. NW contributed to data analysis and revising the manuscript. LT provided a great amount of support and guidance in the research and revised the manuscript substantially.

### Conflict of Interest Statement

The authors declare that the research was conducted in the absence of any commercial or financial relationships that could be construed as a potential conflict of interest.
